# Response of plant life forms and soil physical properties to near-natural restoration measures in alpine grassland, Tibetan plateau

**DOI:** 10.3389/fpls.2024.1504754

**Published:** 2025-01-20

**Authors:** Guoxing He, Xiaoni Liu, Yali Li, Tong Ji

**Affiliations:** ^1^ Key Laboratory of Grassland Ecosystem, Ministry of Education, Pratacultural College, Gansu Agricultural University, Lanzhou, Gansu, China; ^2^ Sino-U.S. Center for Grazing Land Ecosystem Sustainability, Lanzhou, Gansu, China

**Keywords:** near-natural restoration measures, alpine meadows, plant life forms, soil physical properties, soil moisture characteristics

## Abstract

**Introduction:**

Near-natural restoration measures enhance the stability of plant life forms in degraded grasslands, facilitating the natural succession of plant communities.

**Methods:**

In this study, we investigated the effects of three natural restoration measures on the alpine meadows of the northeastern Tibetan Plateau: banned grazing (BG), rest grazing (RG), traditional grazing (TG), and continuous grazing (CG). We utilized redundancy analysis (RDA), variation partitioning(VP), hierarchical partitioning (HP), and partial least squares pathway modeling (PLS-PM) to dissect the quantitative relationships between the distribution of plant life forms and soil physical properties under these restoration measures.

**Results and discussion:**

The results indicated the following: 1) Under each restoration measure, the distribution of life form plants were predominantly characterized by the highest number of hemicryptophytes, followed by geophytes, with the least number of therophytes. We found that the BG treatment had the highest hemicryptophyte height, coverage, aboveground biomass, and importance value, while the CG treatment had the lowest. 2) After near-natural restoration, the soil bulk density (BD) was decreased. The soil moisture characteristics (MC) were increased including soil saturated water content(SSWC), capillary water holding capacity (CWHC), field water capacity (FWC). And capillary porosity (CP) and non-capillary porosity (NP) were increased. 3) VP analysis revealed that MC, BD, and CP together explained 57.4% of the variation in plant life forms communities. 4) The hemicryptophytes benefited from restoration measures and increased CP. In contrast, the decrease in BD negatively affected geophytes. In summary, restoration measures reduce BD by enhancing MC and increasing CP, which affects the distribution of plant life forms. This finding reveals the important role of soil physical properties in plant survival strategies during alpine meadow restoration.

## 1 Introduction

Grasslands are the largest terrestrial biome on Earth, covering about 40% of the land area (Bai and Cotrufo, 2022). Grassland ecosystems are important livestock bases and have a variety of non-material services (Yan et al., 2023). For example, carbon cycling, climate regulation, global biodiversity and human livelihoods (Su et al., 2023). However, nearly half of the world’s grasslands are experiencing degradation due to climate change and overgrazing (Bardgett et al., 2021), with about 5% of the grasslands are severely degraded (Li et al., 2022). Grassland degradation not only leads to the deterioration of the ecological environment but also makes grassland animal husbandry more vulnerable and unstable (Li et al., 2022; Yan et al., 2023). To enhance functions and services of degraded grassland ecosystem, scientists have been proposed near-natural restoration (He et al., 2020). Banned grazing and grazing rest are commonly used near-natural restoration measures (Wang and Wang, 2019; Yang et al., 2020; Zhou et al., 2020). They have been widely applied to degraded grasslands.

Plants flexibly reorganize their life forms to adapt to heterogeneous habitats (Petchey and Gaston, 2002). Plant life forms are categorized types reflected in external morphology, structure, and traits. Life forms were adaptive expressions made by plants in response to specific environments during the long process of natural selection (Mueller-Dombois and Ellenberg, 1974). It is also an important unit of classification for ecological studies. The change of plant life forms can reflect the successional process of the community. This is a crucial metric for evaluating the effects of restoration measures on the community (Whittaker, 1970). The response of plant communities to restoration measures is essentially achieved through the flexible adjustment of life form strategies. This reveals the high adaptability of plants to environmental changes (Niu et al., 2016; Irl et al., 2020). Near-natural restoration measures have dramatically altered the composition and structure of plant communities. This has had a significant impact on the distribution patterns of plant life forms and their overall community dynamics in alpine meadows (Niu et al., 2016). Furthermore, near-natural restoration measures act as a key driver for the natural succession of grassland plant communities, accompanied by complex ecological processes that are centered around the reconfiguration of plant life forms and fluctuations in soil factors (Dai et al., 2021). The relationship between community species composition and environmental factors is a cornerstone of ecological research (Legendre and Fortin, 1989). Although plant life forms have a significant influence on vegetation restoration, litter research has been published on the effects of near-natural restoration on plant life forms.

In previous studies, the focus has been on comparative analyses of vegetation characteristics (e.g., height, coverage, and biomass) and soil properties (e.g., bulk density, water content, and nutrients) under restoration measures (Zhang et al., 2019). Although there has been extensive research on the relationship between vegetation and soil factors, many studies have focused on the relationship between soil factors and plant functional groups, such as grasses, sedges, legumes, and forbs (Li et al., 2018). These studies have focused on the effects of soil chemical properties, while they have solely examined the bulk density and water content of soil physical properties (Li et al., 2018; Zhang et al., 2019). The effects of near-natural restoration on soil moisture characteristics and three-phase ratios have been neglected to some extent.

Notably, plant life forms serve as an important strategy for organisms to adapt to environmental change (Irl et al., 2020). Exploring its relationship with soil water properties. This provides a unique perspective to explain how near-natural restoration measures affect plant community construction. Under restoration measures, plant communities can achieve a fine-tuned selection and adaptive adjustment to changes in soil factors through the reconfiguration and combination of life forms (Mueller-Dombois and Ellenberg, 1974; Chen et al., 2005). This process promotes the establishment of a dynamic feedback mechanism between life forms and soil factors, leading to a state of ecological balance. Life forms can effectively link the ecological interface processes between vegetation and soil. Consequently, considering the soil physical properties and the ‘solid-liquid-gas’ tri-phase composition under near-natural restoration measures holistically can effectively elucidate the impact on plant life forms. The key environmental factors that differentially influence the distribution of various plant life form species under restoration measures are also further identified. This process is crucial for clarifying the unique role of plant life forms in community assembly. Additionally, soil factors, as key indicators of soil quality and ecosystem health, are crucial for the sustainable development of grasslands. Therefore, investigating the patterns of change in plant life forms and soil factors under restoration measures is crucial for devising scientific grassland management strategies.

Alpine grasslands on the Tibetan Plateau are mainly grazed by Tibetan sheep (Ovis aries) and yaks (Bos mutus) and are being degraded by the rapid development of animal husbandry and climate change (Su et al., 2023). To determine the effects of near-natural restoration practices on plant life forms and soil moisture characteristics. We compared banned grazing (BG), rested grazing (RG), traditional grazing (TG), and continuous grazing (CG). We hypothesized that 1) Near-natural restoration resulted in a change in the composition of plant life forms and an increase in the proportion of hemicryptophytes in the community; 2) This change was driven by restoration measures to reduce bulk density (BD) by enhancing soil moisture characteristics (MC) and increasing capillary porosity (CP). The objectives of this study were 1) to determine the response of plant life forms and soil moisture characteristics to near-natural restoration measures in Tibetan plateau; 2) identify the effects of soil moisture characteristics on plant life forms. The application of these results will contribute to the sustainable improvement of the near-natural restoration of grasslands on the Tibetan Plateau.

## 2 Materials and methods

### 2.1 Study site

The experimental plots were located in the Jinqiang River area of Tianzhu Tibetan Autonomous County on the northeastern edge of the Tibetan Plateau, situated between 36°31′-37°55′N, and 102°07′-103°46′E, at an elevation of approximately 2960 m. The region features a cold and humid climate, classifying it as a continental plateau monsoon climate. The average annual temperature was 0.16°C, and the annual precipitation was 416.9 mm. The plant growing season lasts approximately 120 to 140 days. The soil type is alpine chernozem, and the grassland type is an alpine meadow (Li et al., 2018). The dominant species include *Poa pratensis*, *Elymus nutans*, *Kobresia capillifolia*, *Melissitus ruthenica*, *Bistorta vivipara*, and *Potentilla chinensis*, among others.

### 2.2 Experimental design

**Figure 1:**
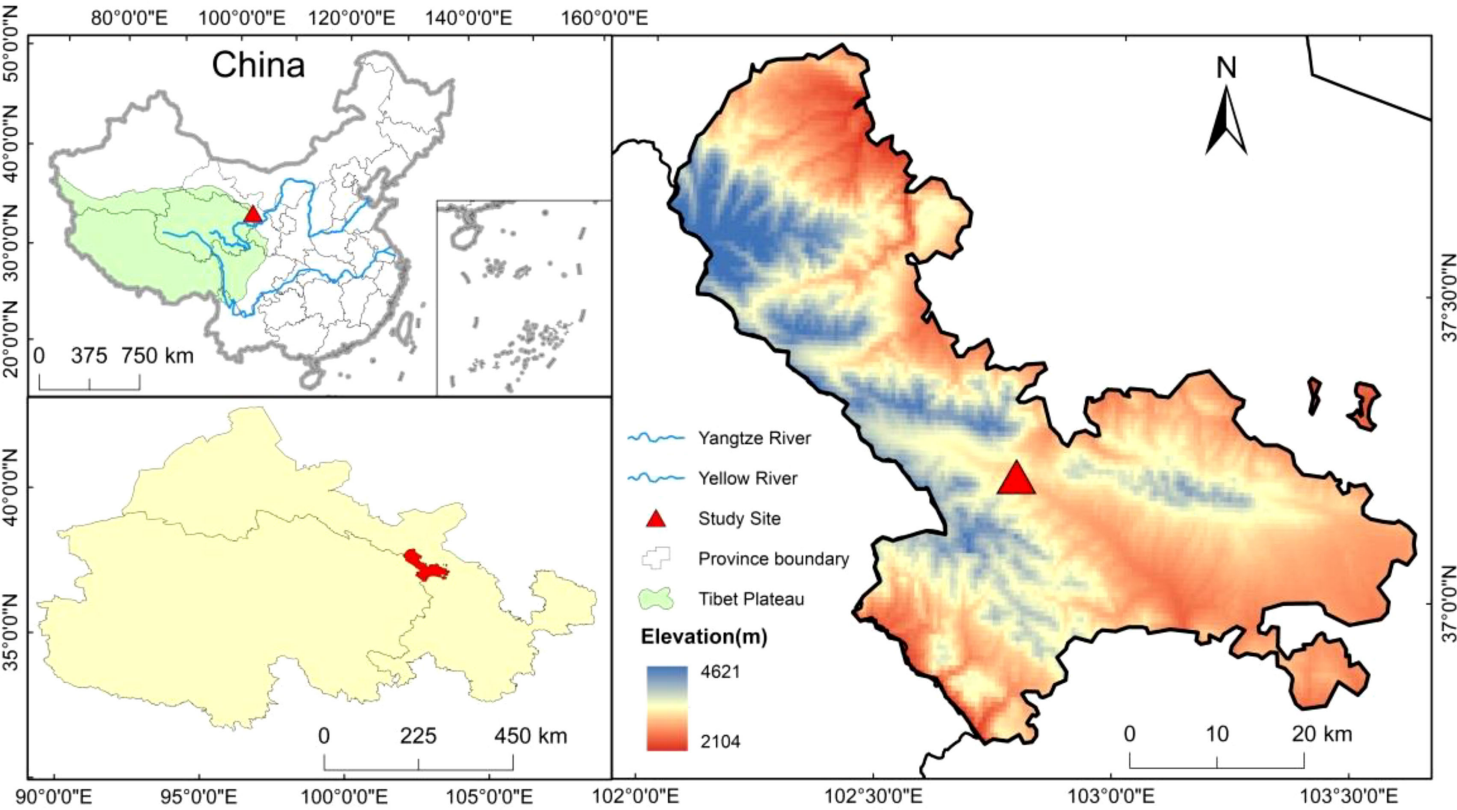
Spatial distribution of sampling sites. Statement. Based on the standard map supervised by the Ministry of Natural Resources of the People’s Republic of china [No. GS (2022) 4316] retrieved from: http://bzdt.ch.mnr.gov.cn/browse.html?picId=%224o28b0625501ad13015501ad2bfc0690%22.

The study selected moderate-deteriorated alpine meadow plots in the northeastern Tibetan Plateau (Li, 1997). They implemented near-natural restoration measures such as BG, RG, and TG since 2003, with CG as the control. A randomized block experiment design was used, with three blocks, each containing the four treatment plots, totaling 12 plots, each measuring 100 m × 100 m. The BG treatment involved no grazing throughout the year, with grazing rates of 0 sheep unit·hm^-2^·a^-1^. The RG treatment involved no grazing from the end of April to the end of September each year, with grazing rates of 3.07 sheep unit·hm^-2^·a^-1^. The TG treatment involved no grazing from the end of June to the end of September each year, with grazing rates of 4.60 sheep unit·hm^-2^·a^-1^. The CG were subjected to grazing throughout the year, with grazing rates of 6.13 sheep unit·hm^-2^·a^-1^. An overview of the plot conditions is shown in **Figure 1**.

### 2.3 Field sampling

Plant community and soil sampling were conducted in mid-August 2022. Within each plot, three 1 m × 1 m quadrats were randomly established, avoiding the edges of the community in the plot, with a minimum distance of 50 m between quadrats. In the end, we obtained 36 quadrats (3 blocks × 4 treatments × 3 quadrats per treatment). To facilitate multivariate analysis, the survey quadrats for plant communities and soil sampling were conducted at the same locations. Soil indicators were measured from soil samples taken at 0-20 cm and 20-40 cm depth layers.

### 2.4 Vegetation characteristics

Using 1 m × 1 m quadrats, a pin was used to make 100 punctures to obtain the coverage of each species within the community; the height of each species was measured randomly 10 times to obtain the height of each species within the community; the density of plants within the quadrat was recorded, and 20 random throws of a circle sample were made near each quadrat to record the frequency of occurrence of each species. The relative values of species height and coverage were calculated using the maximum value standardization method. Finally, one-fourth of the plants in the quadrat were cut off at ground level and placed in a non-woven bag, brought back to the laboratory, killed at 130°C for 30 minutes, and dried at 75°C for 72 hours to determine the aboveground biomass (AGB) (0.01 g). The relative value of biomass was calculated as the sum of the biomass within the quadrat. Species were classified into three life forms (hemicryptophytes, geophytes, therophytes) according to the common classification method of plant life forms (Raunkiaer, 1934).

### 2.5 Soil physical properties

After the vegetation was harvested, undisturbed soil samples from the 0-20 cm (top layer) and 20-40 cm (bottom layer) were collected using a soil ring (100 cm³) for the determination of bulk density (BD), porosity, and moisture characteristics (MC).

#### 2.5.1 The soil moisture characteristics

A 100 cm^3^ (V) soil ring (m_1_) was used to collect the undisturbed soil sample and brought back to the laboratory. The soil ring with the fresh soil (fresh soil + ring weight) was weighed as m_2_. The ring was then placed vertically downward with the perforated end into an iron tray (the depth of the tray being 8-10 cm), and distilled water was slowly added to the tray, causing it to rise slowly to the upper edge of the soil ring. The sample was soaked for 12 h, with additional distilled water added during the soaking process. After saturation, the soil ring was weighed again (m_3_) to measure the soil saturated water content (SSWC). Following the saturation process, the soil ring was carefully transferred, without inverting it, onto a flat tray filled with dry sand. After being placed for 2 h, the soil ring was weighed again (m_4_) to determine the capillary water holding capacity (CWHC). Then, the soil ring was left in place for an additional 24 h. After this period, it was weighed again (m_5_) to measure the field water capacity (FWC) (Cassel and Nielsen, 1986; Chen, 2005; Chen et al., 2019).

After the above weighing (m_5_), take all the soil from the soil ring and placed it into a pre-weighed aluminum box (m_6_). Then placed in a drying oven at 105°C ± 2°C until a constant mass (m_7_) was achieved. The soil bulk density (BD) and soil water content (SWC) were calculated (Blake and Hartge, 1986; Chen, 2005; Chen et al., 2019). The calculation formula is:


$$\text{BD}=\frac{m_{7}-m_{6}}{V}$$

$$\text{SSWC}=\frac{(m_{3}-m_{1})-\left(m_{7}-m_{6}\right)}{m_{7}-m_{6}}$$

$$\text{CWHC}=\frac{(m_{4}-m_{1})-\left(m_{7}-m_{6}\right)}{m_{7}-m_{6}}$$

$$\text{FWC}=\frac{(m_{5}-m_{1})-\left(m_{7}-m_{6}\right)}{m_{7}-m_{6}}$$

$$\text{SWC}=\frac{\left(m_{2}-m_{1}\right)-\left(m_{7}-m_{6}\right)}{m_{7}-m_{6}}$$

The total porosity (TP) of soil is calculated using the formula (Danielson and Sutherland, 1986; Chen, 2005; Chen et al., 2019):


$$\text{TP}=100-\frac{BD}{2.65}\times 100$$

where BD is the soil bulk density and 2.65 is the soil's specific gravity.

The capillary porosity (CP) is calculated as:


$$\text{CP}\;=\frac{\text{CWHC}\times \text{BD}}{water\;density}\times 100$$

where *water density* is 1 g·cm^-3^.

The non-capillary porosity (NP) is calculated by subtracting the capillary porosity from the total porosity:


NP=TP−CP

These calculations provide important information about the soil’s physical properties, which are crucial for understanding water movement and retention in the soil.

#### 2.5.2 The soil ‘solid-liquid-gas’ triphasic system

The calculation of the ‘solid-liquid-gas’ triphasic composition of soil (Danielson and Sutherland, 1986; Chen, 2005; Chen et al., 2019) is as follows:


$$X_{S}=1-TP$$

$$X_{L}=\text{SWC}\times \text{BD}$$

$$X_{G}=TP-\left(SWC\times BD\right)$$

Where:

X_S_ represents the volume percentage of the soil solid phase (%).

X_L_ represents the volume percentage of the soil liquid phase (%).

X_G_ represents the volume percentage of the soil gas phase (%).

### 2.6 Statistical analysis

#### 2.6.1 Height, coverage, density, and importance value of species within life forms

The average height of species within each life form category in each quadrat was used to represent the height of that life form. The sum of the coverage of species within each life form category was used to represent the coverage of that life form. The number of plants of each life form in each quadrat was used to represent the density of that life form. The importance value (IV) of a species was calculated using the formula (Liu et al., 2008):


IV=(A+B+C)/3

Where:

A is the relative height.

B is the relative coverage.

C is the relative biomass.

#### 2.6.2 Redundancy analysis and variation partitioning

In redundancy analysis (RDA), the importance value of each plant was used as the basic data for each species, and a combination matrix of the community was arranged based on life forms, as well as a combination matrix of each life form community was arranged based on species, with both classification units having 36 quadrats.

Soil factors, comprising a total of nine indicators, which are arranged in a soil factor matrix corresponding to the plant quadrats. RDA was used to comprehensively analyze the distribution of communities based on life forms as the basic unit and the relationship between species within each life form and soil factors under near-natural restoration measures (Oksanen et al., 2024). The importance value matrix of plants in each life form was used as the target factor. Variation partitioning (VP) and hierarchical partitioning (HP) (Lai et al., 2022) were used to isolate the effects of moisture characteristics (SSWC, CWHC, and FWC), CP, and BD on plant community patterns. The partial least squares pathway modeling (PLS-PM) was used to explore potential associations between near-natural restoration measures, soil moisture characteristics, CP and BD, and plant life forms. PLS-PM modeling by using the R package “plspm” (Bertrand et al., 2024).

One-way analysis of variance (ANOVA) and least significant difference (LSD) tests were used to assess differences in vegetation characteristics and soil physical properties between different near-natural restoration measures. The statistical significance was *P*< 0.05. The above analysis was performed using R (version 4.2.3) (R Core Team, 2023).

## 3 Results

### 3.1 Comparison of life forms characteristics under different near-natural restoration measures

In this study, the distribution characteristics of plant life forms under four different treatments were investigated. Across all treatments, hemicryptophytes were the most abundant, representing over 65% of the plant species; geophytes were the next most common, with 6, 8, 7, and 6 species respectively under the four treatments; and therophytes were the least abundant (**Table 1**).

**Table 1:** Quantitative distribution of plant life forms under different near-natural restoration measures.

Treat	Number of hemicryptophytes	Percentage/%	Number of geophytes	Percentage/%	Number of therophytes	Percentage/%	Total
BG	25	71.4	6	17.1	4	11.4	35
RG	31	67.4	8	17.4	7	15.2	46
TG	25	67.6	7	18.9	5	13.5	37
CG	21	70	6	20	3	10	30

BG, banned grazing; RG, rest grazing; TG, traditional grazing; CG, continuous grazing.

Overall, different restoration measures have altered the height and coverage of life form species (**Table 2**).

**Table 2:** The comparison on plant life forms height, coverage, density, and aboveground biomass under different near-natural restoration measures.

Treat		BG	RG	TG	CG
Height (cm)	Hemicryptophytes	23.82 ± 2.14^a^	15.82 ± 2.29^b^	11.80 ± 0.48^b^	6.03 ± 0.16^c^
	Geophytes	15.92 ± 1.51^a^	14.42 ± 1.02^a^	10.47 ± 1.14^b^	6.88 ± 0.53^c^
	Therophytes	10.59 ± 1.39^a^	9.76 ± 1.45^a^	3.29 ± 0.58^b^	5.86 ± 0.70^b^
Coverage(%)	Hemicryptophytes	101.89 ± 4.15^a^	101.44 ± 3.33^a^	66.33 ± 2.28^b^	49.56 ± 1.38^c^
	Geophytes	43.44 ± 3.43^ab^	38.22 ± 4.17^b^	50.67 ± 3.90^a^	47 ± 1.26^ab^
	Therophytes	4.22 ± 0.80^b^	7.56 ± 1.23^a^	2.22 ± 0.28^b^	3.89 ± 0.65^b^
Density	Hemicryptophytes	500.33 ± 24.79^a^	529.11 ± 18.97^a^	377.11 ± 22.71^b^	390.44 ± 29.41^b^
	Geophytes	164.56 ± 10.83^b^	269.22 ± 14.60^a^	252.11 ± 24.85^a^	271 ± 13.47^a^
	Therophytes	25.56 ± 4.70^a^	39.89 ± 2.61^a^	19.44 ± 4.28^a^	42.56 ± 16.69^a^
Aboveground biomass(g·m^-2^)	Hemicryptophytes	295.09 ± 5.32^a^	249.59 ± 6.21^b^	153.11 ± 6.60^c^	46.14 ± 2.17^d^
	Geophytes	99.08 ± 7.08^b^	117.46 ± 5.92^a^	128.89 ± 6.94^a^	59.37 ± 3.09^c^
	Therophytes	8.79 ± 0.67^a^	9.23 ± 0.87^a^	4.13 ± 0.80^b^	1.88 ± 0.36^c^

Different lowercase letters indicate significant differences between the four treatments.

BG, banned grazing; RG, rest grazing; TG, traditional grazing; CG, continuous grazing. Different lowercase letters indicate significant differences between the four treatments (*P<* 0.05, *n* = 9)

Hemicryptophytes, geophytes, and therophytes all exhibited the highest species height under the BG treatment, and both the BG and RG treatments were significantly higher than CG (*P*< 0.05). For the three measures, coverage of hemicryptophytes was higher than CG. The coverage of geophytes was not significantly different compared to CG. The coverage of therophytes, RG was significantly higher than CG. The density of hemicryptophyte species was significantly higher in BG and RG compared to CG. The density of geophyte species was significantly lower in BG than in RG, TG, and CG. The aboveground biomass of hemicryptophyte, geophyte, and therophyte species were significantly higher under three measures compared to CG (*P*< 0.05, **Table 2**).

**Figure 2:**
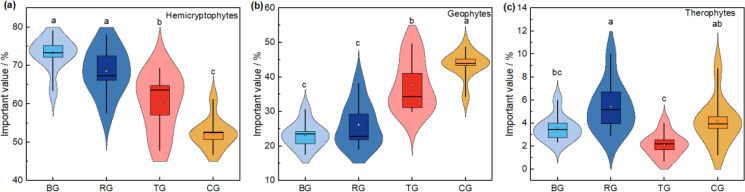
The variation of life forms important value under different near-natural restoration measures. Hemicryptophytes **(A)**, geophytes **(B)**, and therophytes **(C)**.

Further analysis of the importance values of life forms revealed (**Figure 2**) that the importance value of hemicryptophytes was the highest in the BG treatment and significantly higher than that in the TG and CG treatments (*P*< 0.05). The importance value of geophytes was significantly decreased in all three near-natural restoration measures compared to the CG treatment (*P*< 0.05). The importance value of therophytes in the RG treatment was significantly higher than that in the BG and TG treatment.

### 3.2 Comparison of changes in soil physical properties and the ‘solid-liquid-gas’ tri-phase composition under different near-natural restoration measures

**Figure 3:**
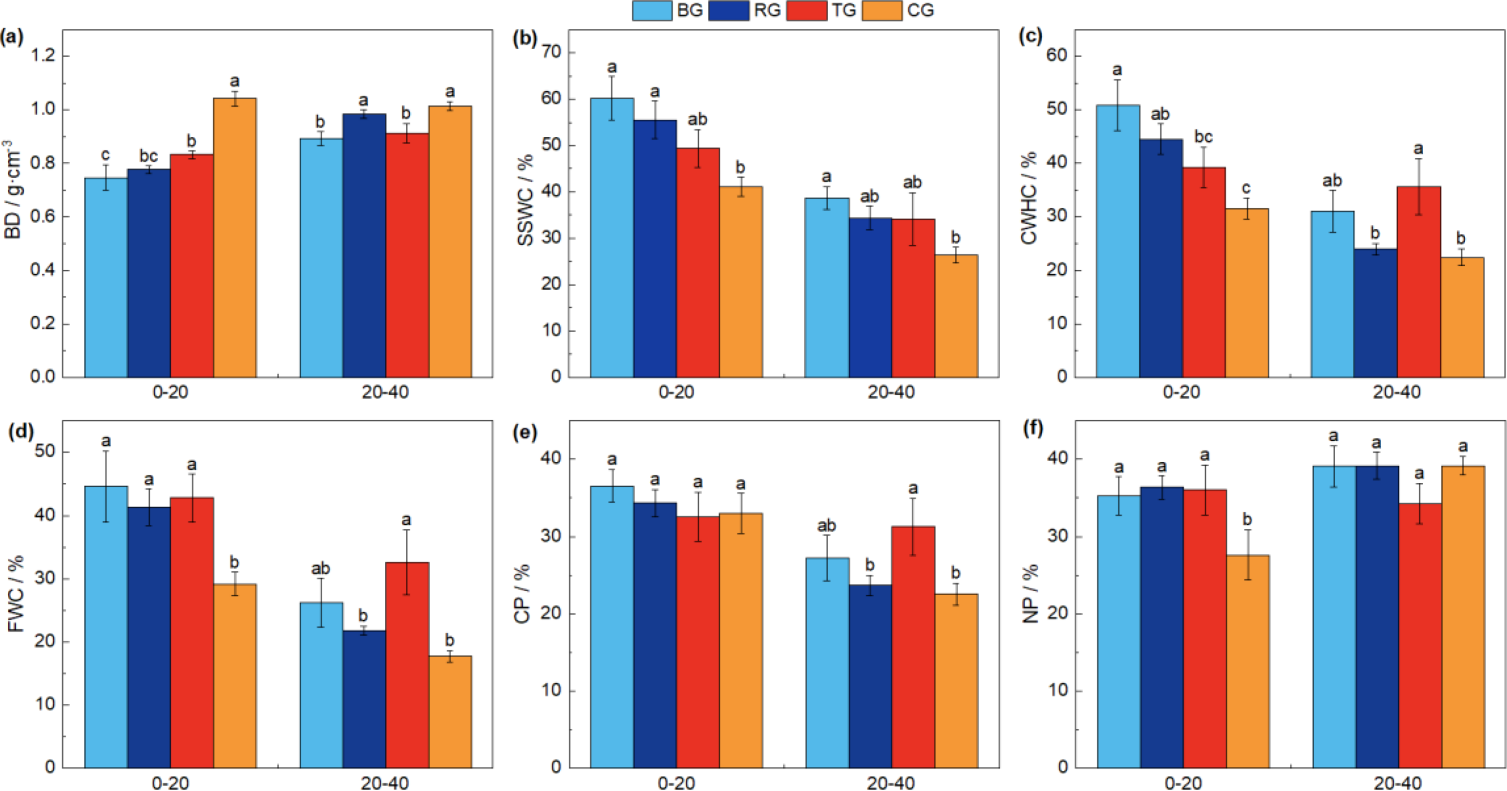
The comparison of soil physical properties under different near-natural restoration measures. Different lowercase letters indicate significant differences between the four treatments (*P< 0.05*, *n* = 9). BD, soil bulk density **(A)**; SSWC, soil saturated water content **(B)**; CWHC, capillary water holding capacity **(C)**; FWC, field water capacity **(D)**; CP, capillary porosity **(E)**; NP, noncapillary porosity **(F)**.

Following near-natural restoration measures, soil physical properties varied with depth, with BD consistently increasing with soil depth. In addition, SSWC, CWHC, FWC, and CP were all greater in the 0-20 cm layer compared to the 20-40 cm layer (**Figure 3**).

The variation in BD across different soil depths followed a pattern where the BG exhibited the lowest values, which were significantly less than CG (**Figure 3A**, *P<* 0.05). The SSWC revealed that BG was significantly higher than CG, with no significant differences among the three restoration measures (**Figure 3B**). In the 0-20 cm soil layer, the CWHC showed that CG was significantly lower than both BG and RG treatments. In the 20-40 cm depth, CG was significantly lower than TG (**Figure 3C**, *P<* 0.05). The FWC in the 0-20 cm layer was significantly enhanced by all three restoration measures compared to CG, and in the 20-40 cm layer, TG was significantly higher than RG and CG (**Figure 3D**, *P*< 0.05).

In the 0-20 cm layer, there were no significant differences in CP (**Figure 3F**) and a significant increase in NP (**Figure 3F**, *P*< 0.05) among the three restoration measures compared to CG. In the 20-40 cm layer, CP exhibited a significant increase in the TG treatment compared to RG and CG (**Figure 3E**, *P*< 0.05). However, there was no significant difference in NP among the three near-natural restoration measures and the CG (**Figure 3F**, *P* > 0.05). These findings underscore the differential impact of restoration measures on soil physical properties at varying depths, which is a critical factor to consider in the ecological restoration of degraded alpine meadows.

**Figure 4:**
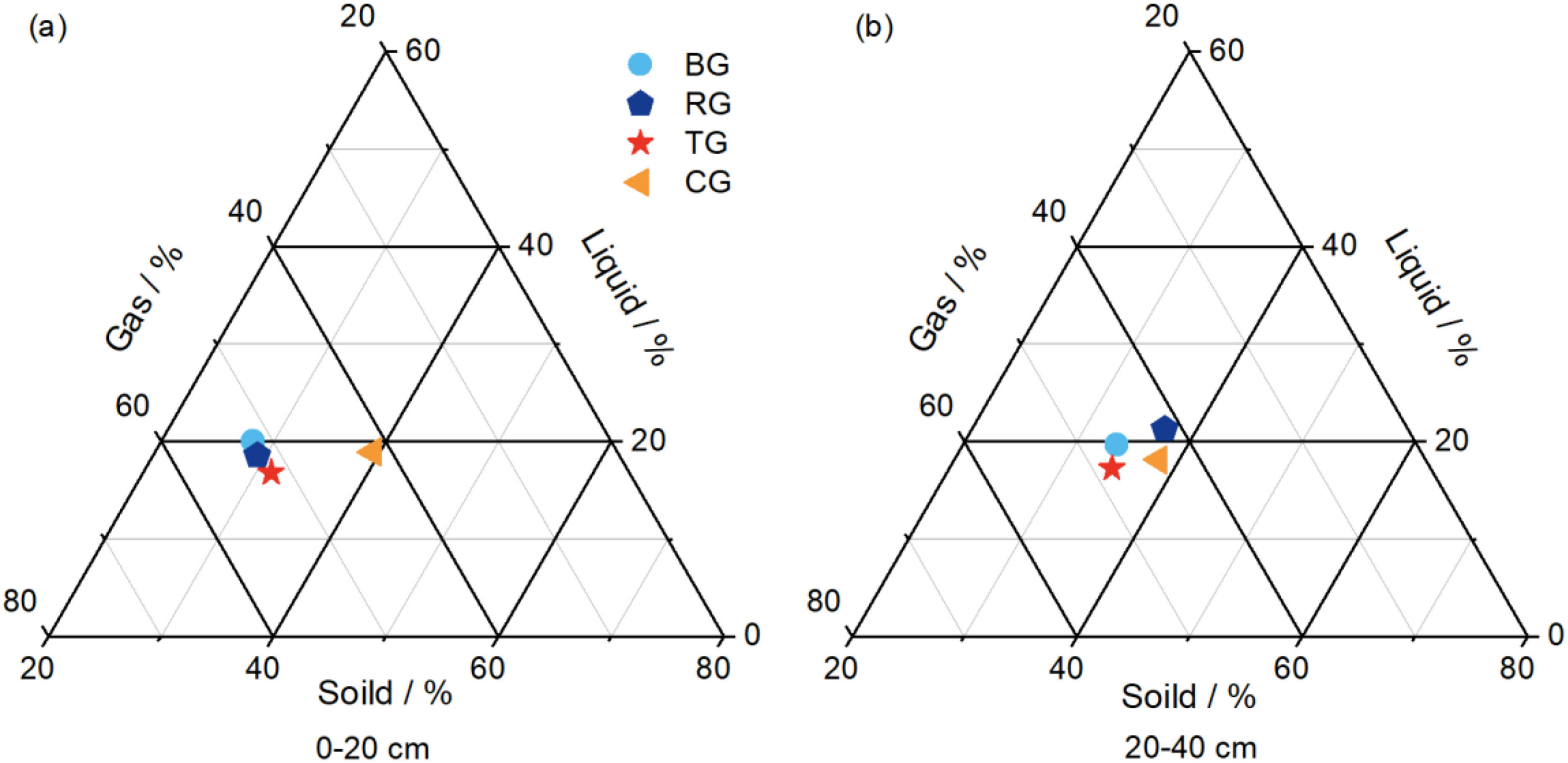
Two-dimensional three-system diagram of soil three phases in 0-20 cm **(A)** and 20-40 cm **(B)** soil depth at different near-natural restoration measures.

As evidenced in **Figure 4**, in the 0-20 cm layer, the solid, liquid, and gas phases were highest in CG, BG, and RG, respectively. In the 20-40 cm layer, the solid phase remained highest in CG, the liquid phase was greatest in RG, and the gas phase by TG. Across all restoration measures, the gas phase predominated, followed by the solid phase, with the liquid phase being the least significant.

### 3.3 Relationships between community composition at the life forms and species levels and soil factors under different near-natural restoration measures

**Figure 5:**
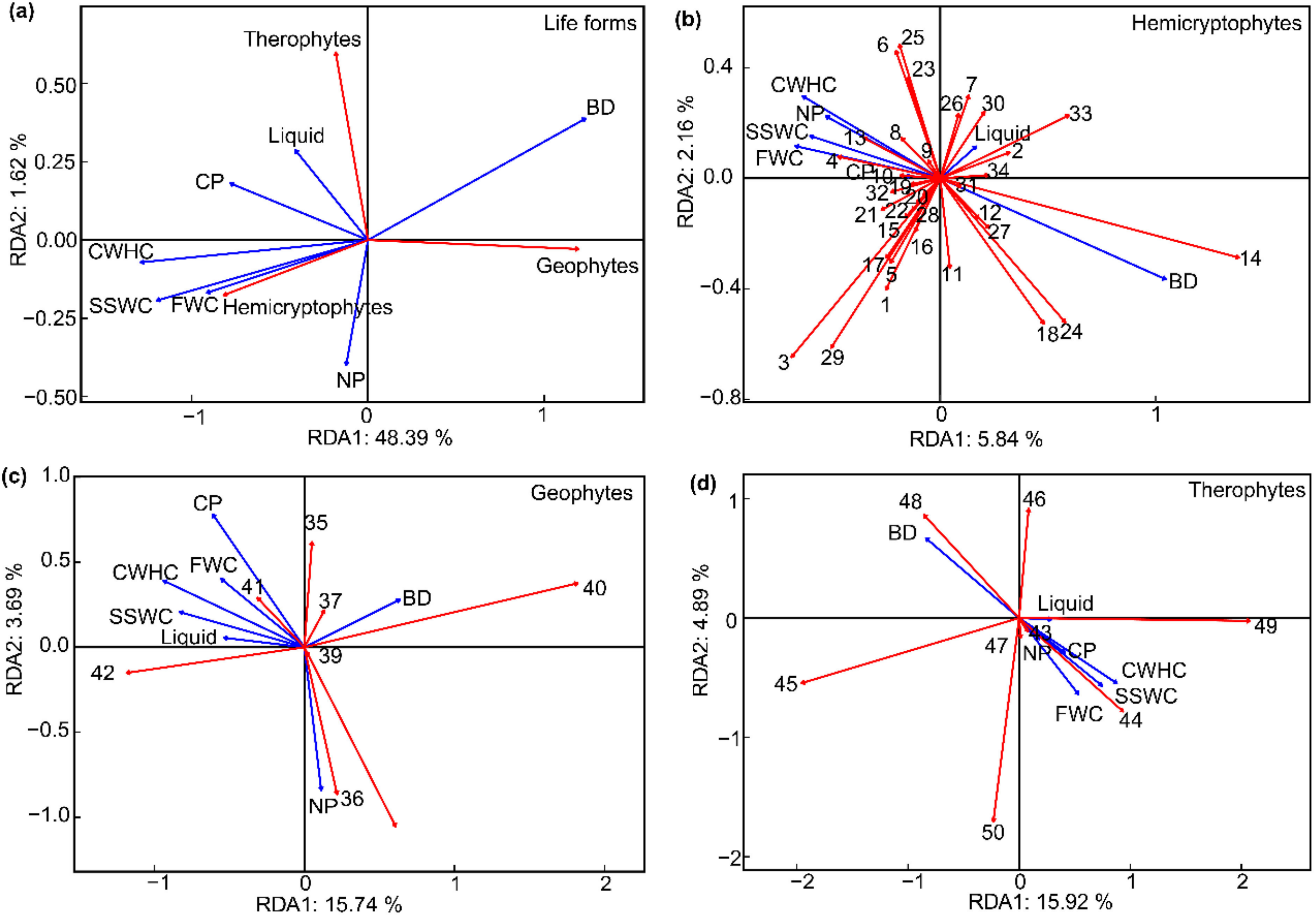
The relationships between life form and plant species composition and soil factor in different near-natural restoration measures **(A)**. BD, soil bulk density; SSWC, soil saturated water content; CWHC, capillary water holding capacity; FWC, field water capacity; CP, capillary porosity; NP, noncapillary porosity; Liquid, soil liquid phase. **(B)** soil factor sorted with hemicryptophytes plants (1-34 for hemicryptophytes plants); **(C)** soil factor sorted with geophytes plants (35-42 for geophytes plants); **(D)** soil factor sorted with therophytes plants (43-50 for therophytes plants).

RDA of the 36 plots across the experimental site revealed that among the different life forms, the CWHC exhibited the strongest positive correlation with hemicryptophytes (**Figure 5A**). Conversely, the BD demonstrated the strongest positive correlation with geophytes.

RDA based on species level revealed the relationship between plant life forms and soil factors (**Figures 5B–D**; **Table 3**). The first axis of the ordination for hemicryptophytes primarily represented BD. BD was positively correlated with the first axis, with a correlation coefficient as high as 0.8. For geophytes, the first axis primarily represented SSWC and CWHC. For therophytes, the first axis was predominantly characterized by CWHC and BD. The CWHC had the strongest correlation with the first axis, at 0.6.

**Table 3:** The correlation coefficients between soil factor and axis under different life form community.

Edaphic variables	Hemicryptophytes		Geophytes		Therophytes	
	RDA1	RDA2	RDA1	RDA2	RDA1	RDA2
BD	0.8	-0.4	0.4	0.3	-0.6	0.6
SSWC	-0.5	0.2	-0.6	0.2	0.5	-0.5
CWHC	-0.5	0.3	-0.7	0.4	0.6	-0.5
FWC	-0.5	0.1	-0.4	0.4	0.4	-0.6
CP	-0.1	0.0	-0.4	0.8	0.3	-0.3
NP	-0.4	0.2	0.1	-0.8	0.1	-0.2
Liquid	0.1	0.1	-0.4	0.1	0.2	0.0

**Figure 6:**
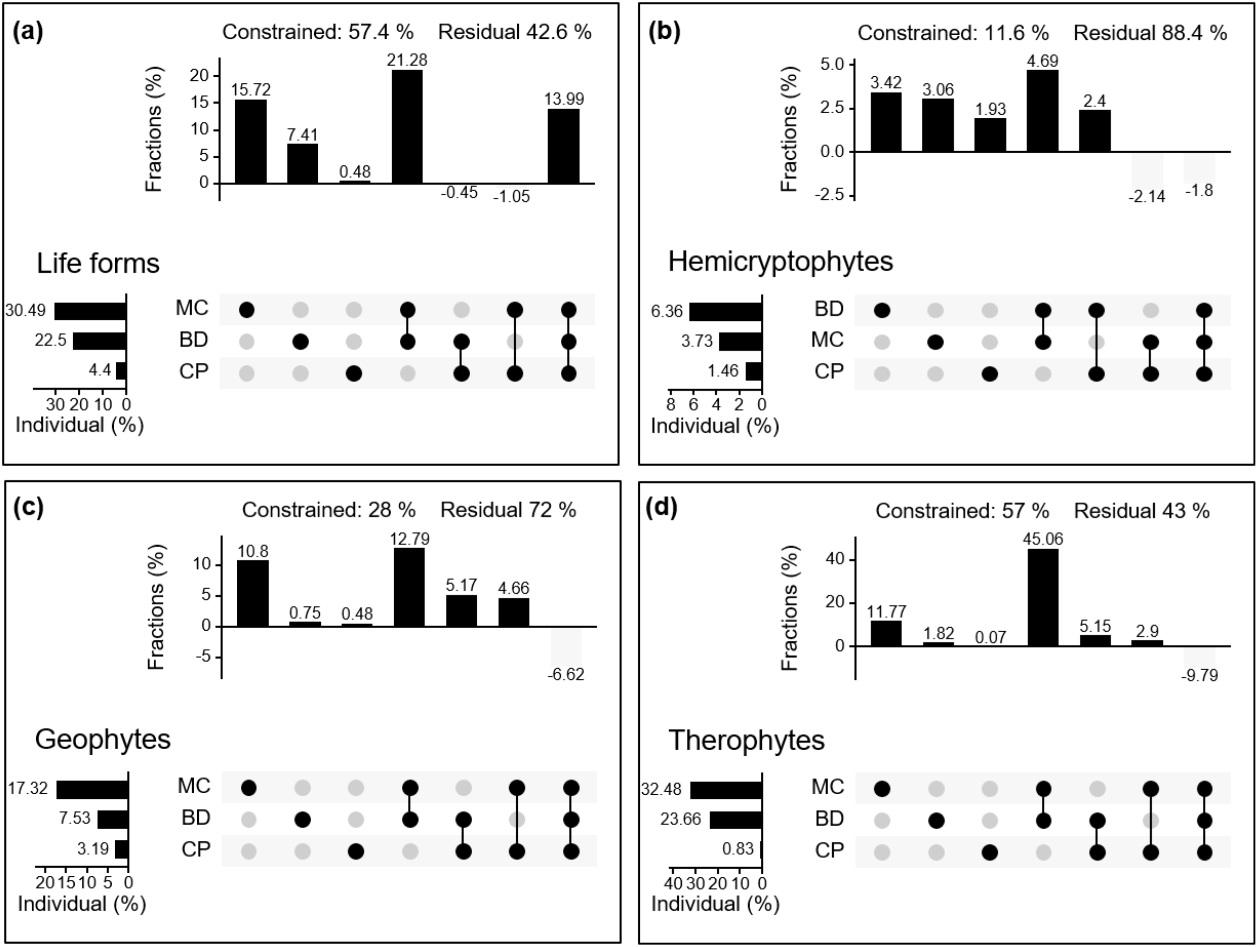
Variation partitioning of moisture characteristics (MC), capillary porosity (CP), and soil bulk density (BD) on plant life forms **(A)** to plant community (life form as the basic unit) and plant species composition **(B–D)**.

Decomposing the contribution of soil physical properties to community composition by plant life forms. The results showed that MC, BD, and CP together explained 57.4% of the variation in plant life form communities. The individual effects of MC and BD were 30.49% and 22.5%, respectively (**Figure 6A**).

In **Figures 6B–D**, it is observed that the environmental factors MC, BD, and CP together explain 11.6% of the variability in the hemicryptophyte community, with BD showing the highest individual effect. For the geophytes community, these three environmental factors together explain 28% of the variability, with MC contributing 17.32% individually. In the therophytes community, MC, BD, and CP together explained 57% of the variance, with the individual effect of MC amounting to 32.48%.

**Figure 7:**
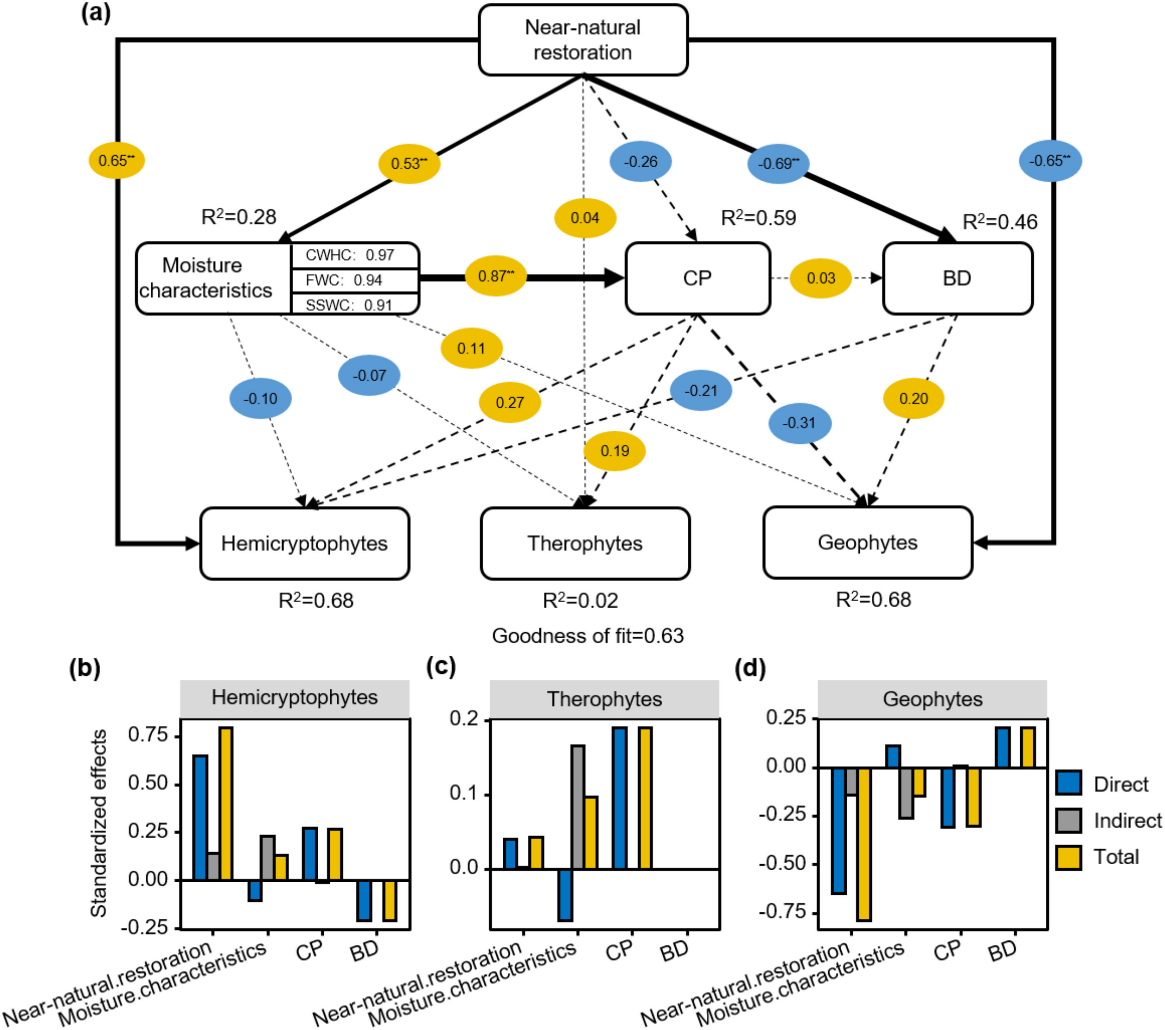
Effect of near-natural restoration measures, moisture characteristics, capillary porosity (CP), soil bulk density (BD) on plant life forms **(A)**; Standardized effects of driving factors on plant life forms **(B–D)**. Yellow and blue circles represent positive and negative effects, while solid and dashed lines indicate significant and insignificant relationships, respectively. *P< 0.05; **P< 0.01.

PLS-PM results showed that hemicryptophytes were positively influenced by restoration measures and CP and negatively correlated with BD. Geophytes had opposite influences to hemicryptophytes (**Figure 7A**). The standardized effect for the drivers indicated that the total effect of restoration measures on hemicryptophytes was 0.79 (**Figure 7B**). The total effect of CP on therophytes was 0.19 (**Figure 7C**). The total effect of BD on geophytes was 0.20 (**Figure 7D**).

## 4 Discussion

### 4.1 Impact of restoration measures on plant life form communities and soil factors

This study demonstrated that plant life forms composition was altered that hemicryptophytes were predominant, with geophytes being the secondary group in alpine meadows after near-natural restoration. This is consistent with our hypothesis. It also reflects the local climate with a brief summer and an extended, severe winter (Duan et al., 2021). Typically, in the harsh cold of high-altitude or high-latitude regions, hemicryptophytes tended to dominate; in cold and wet areas, geophytes were often more abundant (Liu et al., 2003). Guo et al. (2007) discovered that alpine swamp meadows were primarily composed of cold-resistant, wetland-adapted, and mesic hemicryptophytes and geophytes. This study analyzed the role of various life forms in plant communities under different near-natural restoration measures. The results indicated that among the three life forms, hemicryptophytes had a higher number of species and greater importance values. This reflected that hemicryptophytes were a significant component of the plant community across different restoration measures and were the most successful life form in adapting to the harsh winter conditions. This corresponds with the life forms of plant communities in the northeastern edge of the Tibetan Plateau, where winters are severely cold and snowfall is abundant (Elumeeva et al., 2014). Furthermore, this indicated that plant life forms exhibit regular changes across different regions to adapt to varying climatic characteristics (Msadek et al., 2022). Geophytes, though not diverse in species, hold significant importance in plant communities.

In addition, the highest values of height, coverage, aboveground biomass, and importance value were highest in the BG treatment of hemicryptophytes. While CG treatment was the lowest. RG treatment therophytes had the highest importance value. The study revealed that restoration measures directly affected plant communities, leading to changes in the proportional composition of life form characteristics (Verdú et al., 2012). The phenotypic plasticity of life forms objectively expresses the adaptability of plants to restoration measures. Different combinations of life form species correspond to various ecological phenomena, thereby forming distinct life form strategies (Sultan, 1995). This study posited that under the BG restoration measure, the ecological niche of hemicryptophyte species expanded, leading to a rapid increase in their community proportion. The RG and TG restoration measures caused a contraction of the ecological niche for hemicryptophytes, while the community of geophyte species grew rapidly. The implementation of the CG measure restricted the growth of hemicryptophyte species, resulting in significant changes to the overall life form composition of the community. Following near-natural restoration, hemicryptophyte species (e.g., *E. nutans*, *P. pratensis*, *Oxytropis kansuensis*, *P. chinensis*, and *Thalictrum alpinum*) exhibited a declining trend. The geophyte species (e.g., *Leymus secalinus*, *K. myosuroides*, and *B. vivipara*) showed an increasing trend. The therophytes experienced minimal change. The shift in plant life forms is a crucial driver for the structural and functional in the alpine meadow ecosystem. This change is an effect of restoration measures.

Soil physical structural properties, such as BD, moisture characteristics (including SSWC, CWHC, and FWC), and porosity (CP and NP), are vital indicators for assessing the water conservation function of alpine meadows and form the basis for evaluating the sustainable development potential of grassland ecosystems (Dai et al., 2021). This study discovered that near-natural restoration measures significantly altered the soil moisture characteristics of alpine meadows on the northeastern edge of the Tibetan Plateau, particularly in the top layer (0-20 cm) of soil. After near-natural restoration, there was a decrease in BD, while an increase was observed in SSWC, CWHC, FWC, CP, and NP in the degraded alpine meadows. This indicates that near-natural restoration measures can markedly improve the pore conditions of degraded alpine meadows, thereby enhancing the soil’s water conduction capacity and water conservation function, which is consistent with previous research in alpine meadow regions (Yang et al., 2016). This is primarily because, after near-natural restoration, the trampling impact of livestock on the soil has decreased, leading to a loosening of the surface soil structure (Sun et al., 2018). Additionally, the increase in plant coverage has enhanced the interception of settling dust and fine particles by plants, resulting in a higher proportion of fine silt and clay particles in the soil (Yuan et al., 2019). This has led to a reduction in BD, an increase in NP, and an enrichment of CP due to the diverse configurations of plant root systems. Furthermore, nearly two decades of restoration have improved the soil nutrient conditions and increased the organic matter content of the surface soil (Wu et al., 2010), thereby enhancing the soil’s water-holding capacity.

In the study of soil physical structure, a rational composition of the “solid-liquid-gas” tri-phase is considered the cornerstone and core element for the growth and development of plants. Scientific research indicated that an excessively high proportion of the soil solid phase could lead to increased soil compaction, which was detrimental to the extension of plant roots and nutrient uptake. Furthermore, the soil liquid phase, serving as the primary pathway for plants to absorb water and nutrients, was crucial for plant growth, with its appropriate content being vital. Concurrently, the amount of soil gas phase was directly related to the rate of root respiration and nutrient absorption, significantly impacting plant growth and development (Raats, 1984). In this study, the solid phase in the topsoil was highest in the CG, the liquid phase was highest in the BG, and the gas phase was highest in the RG. These findings indicated that after two decades of near-natural restoration, the soil has demonstrated exceptional water retention and storage capabilities, with its physical structure significantly optimized to provide an extremely favorable environment for plant growth. The implementation of restoration measures ensures that the soil provides continuous moisture to the plants, thus promoting their growth.

### 4.2 Impact of soil factors on plant life forms communities under restoration measures

Plant life forms exhibited varying degrees of plasticity in response to restoration measures, which objectively reflected their adaptive capacity to changes in environmental factors (Gómez-Aparicio, 2009; Kose et al., 2021). This study conducted a variance partitioning analysis based on life forms as the fundamental unit of plant communities and found that soil physical explained 57.4% of the variation in plant life form communities. This suggests that soil physical properties drive the reorganization of plant life forms in alpine meadows. Many studies have also shown that changes in soil physical properties are prioritized over others during vegetation restoration (Chai et al., 2019; Duan et al., 2023). After quantitative analysis based on species scale, MC, CP, and BD were found to be important factors determining the distribution pattern of plant life forms (**Figures 6**, **7**). The MC, CP, and BD serve as important structural factors in the physical composition of the soil. Following near-natural restoration, BD significantly decreased, and CP and MC increased. This indicated that after two decades of restoration, the soil demonstrated excellent water-holding capacity. The physical structure of the soil had been significantly optimized, providing an extremely favorable environment for plant growth. Restoration measures ensured the soil’s continuous supply of moisture needed by plants, thereby further enhancing the growth potential of plants (Wu et al., 2024).

In small-scale ecological studies within the same climatic region, restoration measures significantly influenced the dynamics of the soil microenvironment, posing challenges to the composition of plant communities (Brudvig et al., 2017). To ensure the stability of communities was maintained, plant communities had to accurately identify and effectively respond to the most critical stress factors in soil factor, while flexibly adapting to the resulting environmental changes (Mareri et al., 2022). This process involved the recombination of community life forms and the adjustment of species proportions among different life forms as a coping strategy. As key soil properties such as BD, CP, and MC (SSWC, CWHC, and FWC) changed, the community structure exhibited a continuous and predictable pattern of dynamic evolution, deeply reflecting the ecosystem’s adaptability and resilience to external disturbances. For instance, an increase in BD led to soil compaction, which restricted root penetration and growth, affected the absorption of water and nutrients, and simultaneously reduced the soil’s aeration and insulating effect, thereby adversely affecting the normal growth of hemicryptophytes (Assouline, 2006). Soil porosity facilitated the circulation of oxygen in the soil, providing ample oxygen for the respiration of geophytes. Moreover, loose soil, characterized by high porosity, had better insulating effects, which helped to maintain stable soil temperatures around geophytes and accelerated the release and absorption of nutrients (Yu et al., 2018). High CWHC in soil provided more water reserves and better conditions for water absorption for therophytes, satisfying the needs for plant growth and development (Gupta et al., 2015).

HP analysis showed that MC contributed 3.73%, 17.32%, and 32.48% to the distribution of hemicryptophytes, geophytes, and therophytes plant communities, respectively (**Figure 6**). The hemicryptophytes were positively affected by restoration measures and CP, whereas the reduction in BD had a negative effect on hemicryptophytes. Restoration measures affected plant life forms composition by enhanced MC and increased CP, thereby reducing BD. Simultaneously, therophytes were significantly influenced by the MC, indicating that as ‘community compensators’, their growth and distribution were quite sensitive to soil moisture. When sufficient moisture was available, they could grow rapidly and contribute substantially to the grassland biomass (Holmes, 1996). Soil physical properties, including BD, CP, and MC, played a central role in determining the distribution patterns of plant life forms in alpine meadows following different restoration measures. These properties acted as a relatively stable cornerstone within the ecosystem, and changes within the soil often first manifested in the evolution of its physical structural characteristics, subsequently profoundly influencing the patterns of species distribution under restoration efforts (Niu et al., 2019).

Future research should aim to extend the temporal scope of data collection to achieve a more comprehensive understanding of the long-term sustainability of restoration efforts. It is also essential to broaden the scope of our study to encompass a variety of climatic and soil conditions, thereby enhancing the generalizability of our findings. Additionally, incorporating factors such as climate fluctuations, pests’ infestations, and diseases dynamics will provide gain insights into the mechanisms that influence plant life forms and the overall efficacy of restoration strategies under diverse ecological conditions.

## 5 Conclusion

Following near-natural restoration of alpine meadows, the composition of plant life forms has significant changes. The proportion of hemicryptophytes within the community has increased, and geophytes has decreased. Additionally, there is an increase in water holding capacity and a decrease in soil bulk density. The hemicryptophytes benefited from restoration measures and increased capillary porosity. In contrast, the decrease in soil bulk density negatively affected geophytes. Specifically, restoration measures reduce soil bulk density by enhancing water-holding capacity and increasing soil capillary porosity, which affects the distribution of plant life forms. This finding reveals the important role of soil physical properties in plant survival strategies during alpine meadow restoration. Future restoration efforts should prioritize alterations in soil physical properties to enhance the efficiency of vegetation recovery.

## Data Availability

The original contributions presented in the study are included in the article/supplementary material, further inquiries can be directed to the corresponding author.
